# The Regulation of Axon Diameter: From Axonal Circumferential Contractility to Activity-Dependent Axon Swelling

**DOI:** 10.3389/fnmol.2018.00319

**Published:** 2018-09-04

**Authors:** Ana Rita Costa, Rita Pinto-Costa, Sara Castro Sousa, Mónica Mendes Sousa

**Affiliations:** ^1^Nerve Regeneration Group, Instituto de Biologia Molecular e Celular (IBMC) and Instituto de Inovação e Investigação em Saúde, University of Porto, Porto, Portugal; ^2^Instituto de Ciências Biomédicas Abel Salazar (ICBAS), University of Porto, Porto, Portugal

**Keywords:** axon diameter, axonal contractility, axonal tension, axonal cytoskeleton, membrane periodic skeleton

## Abstract

In the adult nervous system axon caliber varies widely amongst different tracts. When considering a given axon, its diameter can further fluctuate in space and time, according to processes including the distribution of organelles and activity-dependent mechanisms. In addition, evidence is emerging supporting that in axons circumferential tension/contractility is present. Axonal diameter is generically regarded as being regulated by neurofilaments. When neurofilaments are absent or low, microtubule-dependent mechanisms can also contribute to the regulation of axon caliber. Despite this knowledge, the fine-tune mechanisms controlling diameter and circumferential tension throughout the lifetime of an axon, remain largely elusive. Recent data supports the role of the actin-spectrin-based membrane periodic skeleton and of non-muscle myosin II in the control of axon diameter. However, the cytoskeletal arrangement that underlies circumferential axonal contraction and expansion is still to be discovered. Here, we discuss in a critical viewpoint the existing knowledge on the regulation of axon diameter, with a specific focus on the possible role played by the axonal actin cytoskeleton.

## Introduction

During development, after reaching their synaptic targets, axons increase their caliber several fold to achieve the large diameters needed for the rapid conduction of action potentials. In the adult nervous system the diameter of axons belonging to different tracts varies by nearly 100-fold (~0.1–10 μm), in accordance with a wide variation in conduction velocity (Perge et al., 2012). When considering a single axon, its diameter can fluctuate in space and time depending on several variables including the distribution of organelles (which is particularly relevant in unmyelinated axons; Greenberg et al., 1990), and activity-dependent mechanisms (Fields, 2011). Mature axons do probably need to further fine-tune their caliber to additional conditions such as deformations imposed by movement (specially in the case of the peripheral nervous system) or induced by axonal degeneration. However, the molecular mechanisms regulating diameter throughout the lifetime of an axon, remain largely elusive.

The axonal cytoskeleton has three major components: microtubules, neurofilaments and actin (Leterrier et al., 2017). Axonal diameter is traditionally regarded as being under the control of the expression and phosphorylation of neurofilaments, the neuron-specific intermediate filaments. Loss of all axonal neurofilaments results in smaller caliber myelinated axons with slower conduction velocities (Ohara et al., 1993; Sakaguchi et al., 1993; Zhu et al., 1997; Kriz et al., 2000). C-terminal domain neurofilament phosphorylation is a key component of radial growth (de Waegh et al., 1992) as it aligns and bundles neurofilaments and extends their sidearms (Leterrier et al., 1996) promoting the crosslinking among neurofilaments and other cytoskeletal components (Gotow et al., 1994). This sequence of events leads to an increase in inter-filament spacing and thereby to the increase of axon diameter (Nixon et al., 1994). Assembly of compact myelin is a key driver in the initiation of radial axonal growth as signaling from myelinating glia triggers the above events (de Waegh et al., 1992). Accordingly, unmyelinated regions of a given axon have smaller diameters, containing less phosphorylated, and more compact neurofilaments (Hsieh et al., 1994).

In small caliber axons that express low levels of neurofilaments, microtubule organization contributes to the regulation of axon diameter (Friede and Samorajs, 1970; Alfei et al., 1991). In arthropods, that totally lack neurofilaments (Benshalom and Reese, 1985; Goldstein and Gunawardena, 2000) axon diameter is also controlled by microtubule-dependent mechanisms. In *Drosophila*, two giant Ankyrin2 isoforms (Ank2-L and Ank2-XL) and the microtubule-associated protein 1B (MAP1B) homolog Futsch form an evenly spaced, grid-like membrane-associated microtubule-organizing complex that determines axon diameter in the absence of neurofilaments (Stephan et al., 2015). Interestingly, a similar grid-like microtubule organization has been observed in mammalian retinal axons, where microtubule tract number correlates with axon diameter (Hsu et al., 1998; Perge et al., 2012). On a different note, and further supporting the significant role of microtubules in the control of axon shape and diameter, axon degeneration is often accompanied by focal axonal enlargements—axonal swellings—that have been mainly related to the disorganization of the microtubule cytoskeleton (Saxena and Caroni, 2007).

Although it has long been known that axons are contractile along their longitudinal axis (Dennerll et al., 1988; George et al., 1988; Rajagopalan et al., 2010) only recent emerging evidence support that axons may also exert contractility/tension along their circumferential axis (Fan et al., 2017). The role of the axonal cytoskeleton, namely of actin microfilament organization and dynamics in the regulation of axonal tension, is beginning to come to light. This has been made possible by the development of novel actin probes, super-resolution microscopy and state-of-the-art live imaging that allowed unveiling previously undiscovered axonal actin structures (Leterrier et al., 2017).

## Regulation of Axon Diameter by the Membrane Periodic Skeleton: The Role of Adducin

**Figure 1:**
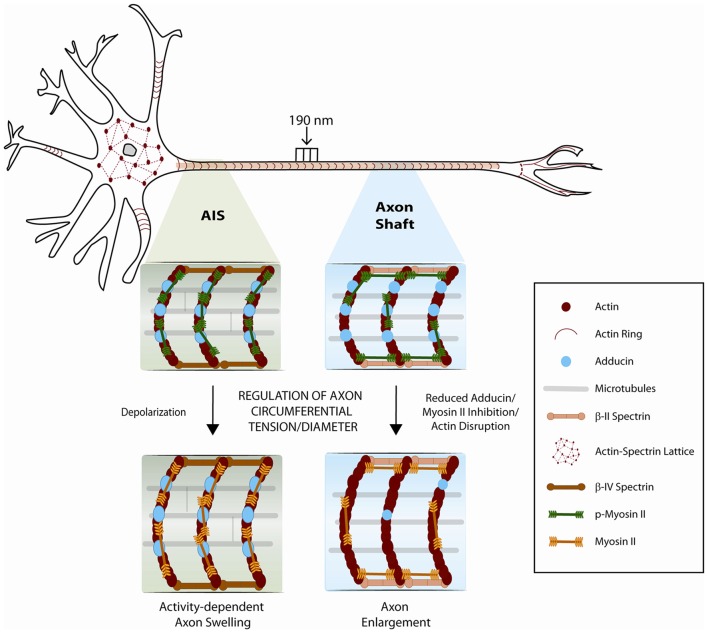
Schematic representation of the neuronal cytoskeleton. The role of the membrane periodic cytoskeleton and of myosin-II in the control of the axonal circumferential contractility is emphasized. In mature neurons, a 1D membrane periodic skeleton is present not only in axons but also in a fraction of dendrites. In contrast, in the somatodendritic compartment, an actin-spectrin based 2D polygonal lattice is formed, resembling the expanded erythrocyte membrane skeleton. Phosphorylated myosin light chain is highly enriched at the axon initial segment, where it may participate in the regulation of axon diameter during action potential firing. In the axon shaft, circumferential and longitudinal axon tension may also result from the regulation of an actomyosin network that remains to be solved at the structural level. Adducin, and additional actin-binding proteins that might be associated to axonal actin rings, may further contribute to fine-tune axon diameter.

It is now recognized that the mature axon shaft has two distinct actin cytoskeletal arrangements: (i) a submembranous stable actin-spectrin network (the membrane periodic skeleton), composed of actin rings regularly spaced by spectrin tetramers approximately every 190 nm (Xu et al., 2013; Figure 1), and (ii) deep dynamic actin trails, consisting of focal actin hot spots and elongating actin polymers along the shaft (Ganguly et al., 2015; Roy, 2016). Work from our group and others demonstrated that the membrane periodic skeleton is present in every neuron type, ranging from central to peripheral nervous system neurons (D’Este et al., 2015; He et al., 2016; Leite et al., 2016), including both excitatory and inhibitory neurons (D’Este et al., 2016; He et al., 2016), being observed in fixed and live cells, and in brain tissue sections, without substantial differences between unmyelinated and myelinated axons (Xu et al., 2013; Lukinavičius et al., 2014; Zhong et al., 2014). In hippocampal neuron cultures, the membrane periodic skeleton emerges early during axon development, after axon specification, and propagates from proximal regions to distal ends of axons, spanning almost the complete axon shaft of mature neurons (Han et al., 2017). Once matured, the structure is thought to be highly stable, with slow turnover of its components (Zhong et al., 2014). Recent data obtained using hippocampal neuron cultures support that the subcortical neuronal cytoskeleton is differentially regulated in the different neuronal compartments (Han et al., 2017; Figure 1). In mature neurons, a 1D membrane periodic skeleton is present not only in axons but also in a substantial fraction of dendrites. However, in dendrites, this structure develops slower and forms with a lower propensity than in axons (Han et al., 2017). In contrast, in the somatodendritic compartment, an actin-spectrin based 2D polygonal lattice is slowly formed, resembling the expanded erythrocyte membrane skeleton (Han et al., 2017). *In vitro*, in hippocampal neurons, the average dendritic diameter is higher than that of axons, which led to the hypothesis that the lower propensity of 1D lattice formation in dendrites could stem from their larger diameter (Han et al., 2017). Yet, further analyses showed little dependence between dendrite diameter and formation of the membrane periodic skeleton. The authors did however not exclude the hypothesis that in very wide neurites, in which the membrane is locally nearly flat, the actin-spectrin network may adopt a different structural form (Han et al., 2017).

The discovery of the membrane periodic skeleton opened new perspectives on how the actin cytoskeleton might support the neuronal architecture and function. Although its assembly mechanism and function remain largely elusive, the actin-spectrin network may provide mechanical support for the long and thin structure of axons which can be particularly vulnerable to tissue pressure. Supporting this notion, in *C. elegans*, deletion of beta spectrin leads to axon breakage upon movement of the worm (Hammarlund et al., 2007).

In axonal actin rings, the actin-binding protein adducin caps the barbed end of actin filaments (Xu et al., 2013). Hence, in the initial model of the organization of the membrane periodic skeleton, actin rings were proposed to be made of short filaments (Xu et al., 2013), likely assisted by additional actin-binding proteins (Figure 1). Work from our group has further demonstrated that *in vitro*, in the absence of adducin, actin rings present an increased diameter which *in vivo* results in progressive axon enlargement, followed by axon degeneration and loss (Leite et al., 2016; Figure 1). These findings support that the capping activity of adducin is required to maintain axon diameter. Of note, *in vitro*, the actin ring diameter of both WT and α-adducin KO neurons narrowed over time. This supports that actin filaments that compose the axonal actin rings are more dynamic than initially suggested, being able to adapt to variations in axon diameter. Analysis throughout time of F-actin content in actin rings supported that axon constriction occurs with bundling and condensation of actin filaments within rings (Leite et al., 2016). Since both WT and α-adducin KO neurons decrease actin ring diameter over time in culture, the dynamics of the submembranous actin-spectrin network is probably regulated by additional actin-binding proteins. Variations in axon diameter also raise exciting questions on the arrangement of spectrin tetramers within the membrane periodic skeleton. Future experiments should address how spectrin density i.e., its lateral spacing within axons is adjusted when variations of axon caliber occur.

There are several resemblances between the actin-spectrin lattice in neurons and erythrocytes. In red blood cells, spectrin, actin and associated proteins form a cortical cytoskeleton that confers strength and elasticity (Mohandas and Gallagher, 2008). The lack of adducin results in misshaped erythrocytes that are less resistant to mechanical stress, including osmotic pressure (Gilligan et al., 1999; Robledo et al., 2008). Recently, using super-resolution microscopy, it has been demonstrated that red blood cells contain bipolar filaments of non-muscle myosin II associated to the membrane skeleton (Smith et al., 2018). Non-muscle myosin II-mediated contractility is a highly conserved mechanism for generating mechanical forces and translocation of the actin cytoskeleton in non-muscle cells (Vicente-Manzanares et al., 2009). Myosin II is a hexameric molecule consisting of a dimer of heavy chains, two regulatory light chains and two essential light chains (Vicente-Manzanares et al., 2009). Similarly to smooth muscle myosin, non-muscle myosin II is activated by phosphorylation of myosin light chain, either by an initial single phosphorylation at Ser19 or by sequential phosphorylation of Thr18 and Ser19 (Vicente-Manzanares et al., 2009). Myosin light chain phosphorylation activates both the contractile ATPase activity of non-muscle myosin II and the assembly of myosin filaments needed to coordinate force generation (Vicente-Manzanares et al., 2009). In red blood cells, the non-muscle myosin II light chain is phosphorylated, indicating active regulation of its motor activity and filament assembly. Inhibition of non-muscle myosin II with blebbistatin, an inhibitor of its motor activity (Kovács et al., 2004), enhances membrane deformability and decreases membrane tension. This supports a role for myosin II-mediated contractility in promoting membrane stiffness and maintaining red blood cell shape. Similarly to erythrocytes and to actin rings that exist in other biological contexts, the membrane periodic skeleton may have a more dynamic nature than initially anticipated, fine-tuning the circumferential contractility/tension of the axon shaft (Leite and Sousa, 2016). One can also not exclude the participation of the deep axonal actin cytoskeleton i.e., the actin hot spots and actin trails in the regulation of the axonal circumferential and/or longitudinal tension.

## Mechanisms Regulating Axonal Longitudinal and Circumferential Contractility

Unperturbed neurons maintain an intrinsic longitudinal rest tension along their axons both *in vitro* (Heidemann and Buxbaum, 1990) and *in vivo* (Siechen et al., 2009). *In vitro*, when tension is increased, a rapid axon elongation takes place (Bray, 1984; Pfister et al., 2004) in a process known as “axon stretch growth” (reviewed in Smith (2009)). In the case of embryonic rat dorsal root ganglia neurons, exposure to mechanical tension enables elongation at a rate of 8 mm/day resulting in axons a thousand times their original length (Pfister et al., 2004). Evidence of severe axon stretch growth is found *in vivo* in nature and is well pictured by the rapid expansion in the blue whale’s spine axons that grow at a rate higher than 30 mm/day (Smith, 2009). In contrast, in response to loss of tension, axons contract as is the case after resection or surgical incision (Shaw and Bray, 1977; Joshi et al., 1985; George et al., 1988; Gallo, 2004). This longitudinal contraction eventually leads to the re-establishment of the axonal rest tension. The actomyosin network has emerged as a central regulator of the intrinsic axial tension (i.e., axonal contractility along the longitudinal direction) both *in vitro* (Dennerll et al., 1988; Lamoureux et al., 1989) and *in vivo* (Siechen et al., 2009; Xu et al., 2010; Tofangchi et al., 2016; Figure 1).

*In vivo*, in embryonic *Drosophila*, when motor neuron axons are slackened mechanically, axons shorten within 2–4 min and restore their straight configuration (Tofangchi et al., 2016). This longitudinal contractility decreases dramatically after myosin II knockdown and inhibition, namely by using ML-7, an inhibitor of myosin light chain kinase (Saitoh et al., 1987), and Y-27,632 (Uehata et al., 1997), an inhibitor of Rho-associated protein kinase (ROCK). Further supporting the involvement of myosin II in longitudinal axonal contractility, disruption of actin filaments in embryos treated with cytochalasin D and latrunculin A (Spector et al., 1989), significantly impaired axonal contraction. Interestingly, the rate of contraction is faster when microtubules are disrupted by either nocodazole or colchicine (Tofangchi et al., 2016). Using trypsin-mediated detachment in chick embryo DRG neuron cultures, as an alternative model to evaluate axonal longitudinal contraction (Mutalik et al., 2018), blebbistatin inhibits axon straightening upon trypsin-induced de-adhesion. In contrast to the *Drosophila* model, in chick DRG neurons nocodazole reduces axonal contraction (Mutalik et al., 2018). As such, the role of microtubules in longitudinal axonal contraction awaits further clarification.

In addition to longitudinal contractility, using confocal microscopy and spatial light interference microscopy, it has been recently shown that *Drosophila* axons also actively maintain contractility/tension along the circumferential axis (Fan et al., 2017). Similarly to axial tension, circumferential tension is also regulated by myosin-II (Figure 1). Increased axonal diameter is generated by disruption of actin filaments or by myosin II inhibition using either ML-7 or the ROCK inhibitor 27632 (Fan et al., 2017). These results suggest that the actomyosin machinery is contractile along the circumferential direction of axons such that the relaxation of tension results in increased axonal diameter. The authors also raised the hypothesis that circumferential tension applies a compressive force on microtubules, and that the force balance between cortical actin and microtubule results in an equilibrium diameter of the axon. Accordingly, a decrease in axon diameter was observed when microtubules were disrupted with nocodazole or colchicine. In this context, it is interesting to note that the membrane periodic skeleton is thought to interact with axonal microtubules (Zhong et al., 2014; Qu et al., 2017). Additionally, in *Drosophila* neurons, formins (actin nucleators that crosslink actin and microtubules) were shown to contribute to the actin-spectrin network (Qu et al., 2017). As such, one cannot exclude that cytoskeleton crosslinkers may also be involved in modulating axon diameter. An additional layer of regulation may be provided by Ca^2+^ levels. Given that Ca^2+^-calmodulin controls adducin (Gardner and Bennett, 1987; Kuhlman et al., 1996) and non-muscle myosin II activity (Somlyo and Somlyo, 2003), it is plausible that changes in axon diameter might be regulated by Ca^2+^ influx.

Overall, a mechanism in which longitudinal and circumferential axonal tension are coupled, is supported by recent data (Fan et al., 2017), as they share similar time constants of evolution; i.e., the times to generate longitudinal tension and to contract the diameter to their respective steady values are similar. However, the cytoskeletal arrangement that underlies axial and circumferential axonal contractility, and their coupling, remains to be resolved.

## Activity-Dependent Changes of Axon Diameter: Axonal Swelling During Action Potential Generation

Several data support that axon diameter is dynamic and regulated by activity-dependent mechanisms. In fact, nerves and axons from different species including giant axons of squid (Iwasa and Tasaki, 1980; Tasaki and Iwasa, 1982), crayfish (Hill et al., 1977) and cuttlefish (Hill, 1950), the garfish olfactory nerve (Tasaki et al., 1989; Tasaki and Byrne, 1990), the bullfrog olfactory bulb (Tasaki and Byrne, 1988), dorsal root ganglia and spinal cord (Tasaki and Byrne, 1983), are known to swell during the production of an action potential (reviewed in Fields (2011)). In the squid giant axon, swelling starts nearly at the onset of the action potential and the peak of swelling during excitation coincides accurately with the peak of the action potential recorded intracellularly (Iwasa and Tasaki, 1980). Of note, concurrently with axon swelling, longitudinal shortening of nerve fibers occurs (Tasaki and Iwasa, 1982; Tasaki et al., 1989). This observation points towards the similarities between the mechanical changes that occur in the muscle and those that take place in the nerve (Tasaki and Byrne, 1990).

Recently, the inability of conventional light microscopy to resolve thin unmyelinated axons, which can have diameters well below 200 nm i.e., below the limit of resolution of an optical microscope, has been overcome by the development of super-resolution microscopy. In agreement with the above seminal studies, using time-lapse STED microscopy in mouse brain slices, axons were shown to swell after high-frequency action potential firing (Chéreau et al., 2017). In these settings, whereas synaptic boutons underwent a rapid transient enlargement that decayed, the axon shaft showed a more delayed and progressive increase in diameter, swelling gradually over the duration of the experiment (approximately 1 h). Electrophysiological experiments revealed a short phase of slowed down action potential conduction linked to the transient enlargement of the synaptic boutons (Chéreau et al., 2017). This initial phase was followed by a sustained increase in conduction speed when the axon shaft widened. In summary, increasing axon diameters accelerated action potential conduction along the axons. This finding is in line with cable theory (Goldstein and Rall, 1974) as axons of increased diameter have less internal electrical resistance, which facilitates the spread of action potential. This study shows that activity-dependent changes in the nanoscale axon morphology modify the speed of action potentials along hippocampal unmyelinated axons, revealing a new layer of complexity for the regulation of axon physiology. The authors raised the hypothesis that rapid redistribution and/or *de novo* synthesis of membrane might be the source of the activity-dependent net enlargement of axons.

Lately, new insights have been provided for the physical mechanisms that may account for the increase in axon diameter during action potential firing (Berger et al., 2018). The axon initial segment, located in the proximal axon of multipolar neurons, is the region where action potentials are generated (Ogawa and Rasband, 2008). This region is crucial for fine-tuning neuronal excitability as its structural properties, including length and/or location relative to the soma, change in an activity-dependent manner (Yamada and Kuba, 2016). The mechanisms that underlie the assembly of the axon initial segment in the proximal axon and its plasticity are still poorly understood. Recently, it has been demonstrated that phosphorylated myosin light chain is highly enriched in the axon initial segment (Berger et al., 2018) where its contractile activity may function as the molecular mechanism regulating activity-dependent changes of axon diameter (Figure 1). Interestingly, myosin light chain was shown to be rapidly lost during depolarization, via Ca^2+^-dependent mechanisms, destabilizing actin and thereby providing a mechanism for activity-dependent structural plasticity of the axon initial segment (Berger et al., 2018). Using STORM nanoscopy, the authors proposed that activated phospho-myosin light chain associates with the periodic actin cytoskeleton forming actomyosin rings at the axon initial segment, and raised the hypothesis that myosin II filaments are oriented parallel to the actin rings. It is however interesting to speculate the possible existence of an alternative model, in which myosin filaments might not be exclusively oriented in parallel to actin rings, thus providing the additional control of longitudinal axonal contractility (Figure 1).

## Conclusion and Perspectives

The cytoskeletal arrangement that regulates circumferential axonal contraction and expansion is just starting to be unveiled. Understanding its structure is likely to be a challenging enterprise as it will most probably rely on the simultaneous detection of multiple axonal components by super-resolution microscopy. Additionally, although axons are generally depicted as straight regular structures, even *in vitro* their courses can be tortuous with complex twisting that may hamper withdrawing straightforward conclusions. If, as suggested by an emergent body of literature, an actomyosin network participates in the fine control of axon diameter, a possible interplay between the axonal subcortical cytoskeleton and the deep axonal actin filaments, as putative anchors of myosin filaments, may need to be explored. This is certainly a very exciting field of research that will further the notion that the cytoskeleton in the axon shaft is likely to be much more dynamic than initially expected.

## Author Contributions

MS, AC, RP-C and SS wrote the manuscript.

## Conflict of Interest Statement

The authors declare that the research was conducted in the absence of any commercial or financial relationships that could be construed as a potential conflict of interest.
